# Long-Term Infectious and Noninfectious Outcomes of Monthly Alemtuzumab as a Calcineurin Inhibitor- and Steroid-Free Regimen for Pancreas Transplant Recipients

**DOI:** 10.1155/2020/8883183

**Published:** 2020-10-09

**Authors:** Adam Kaplan, Jo-Anne H. Young, Raja Kandaswamy, Danielle Berglund, Bettina M. Knoll, Gretchen Sieger, Winston Cavert, Arthur Matas, Karam M. Obeid

**Affiliations:** ^1^University of Minnesota, School of Public Health, Division of Biostatistics, Minneapolis, Minnesota 5455, USA; ^2^University of Minnesota, Department of Medicine, Division of Infectious Diseases and International Medicine, Minneapolis, Minnesota 55455, USA; ^3^University of Minnesota, Department of Surgery, Minneapolis, Minnesota 55455, USA; ^4^University of Minnesota, Clinical and Translational Science Institute, Minneapolis, Minnesota 55455, USA; ^5^New York Medical College, Department of Medicine, Valhalla, New York 10595, USA

## Abstract

Multiple doses of alemtuzumab for immunosuppressive therapy of patients with hematologic malignancies and hematopoietic stem cell transplant have been associated with a high rate of infection. In transplantation, limited alemtuzumab dosing has been successfully used as induction immunosuppression. The effect of multiple doses of alemtuzumab, used as maintenance therapy to minimize steroid and/or calcineurin inhibitor toxicity in solid organ transplant recipients, is unknown. We evaluated the infectious and noninfectious outcomes of 179 pancreas transplant recipients treated with alemtuzumab for induction and maintenance therapy (extended alemtuzumab exposure (EAE)) from 2/28/2003 through 8/31/2005, compared with 159 pancreas transplant recipients with standard induction and maintenance (SIM) therapy performed before (1/1/2002 until 12/31/2002) and after (1/1/2006 until 12/31/2006) the implementation of EAE. EAE was associated with higher risk of overall infections (hazard ratio (HR) 1.33 (1.06–1.66), *P*=0.01), bacterial infections (HR 1.33 (1.05–1.67), *P*=0.02), fungal infections (HR 1.86 (1.28–2.71), *P* < 0.01), and cytomegalovirus infections (HR 2.29 (1.39–3.77), *P* < 0.01). In addition, EAE was associated with higher risk of acute cellular rejection (HR 2.09 (1.46–2.99), *P* < 0.01). In conclusion, while a limited alemtuzumab dosing is safe and effective for induction therapy in pancreas transplantation, EAE combined with steroid and calcineurin minimization is associated with a high risk of infectious complications and acute cellular rejection.

## 1. Introduction

Alemtuzumab (Campath-1H, MabCampath®) is a humanized monoclonal antibody directed against CD52, a glycoprotein expressed on circulating T- and B-lymphocytes, monocytes, macrophages, dendritic cells, and NK cells [1, 2]. Alemtuzumab is used for the treatment of hematologic malignancies (HM) and the prevention of graft versus host disease (GVHD) in hematopoietic stem cell transplant (HSCT) recipients [1–4]. However, with the treatment of HM and HSCT, infectious complication rates are proportionately related to the number of cumulative doses of alemtuzumab [3–6].

In solid organ transplantation (SOT), since 1984, calcineurin inhibitors (CNI) have been the backbone of the maintenance immunosuppressive (IS) therapy; they are, however, associated with multiple adverse events including nephrotoxicity. Induction IS therapy with limited alemtuzumab dosing (either a single 30 mg single-dose or a total of 40 mg in two divided doses) has been successfully used with or without CNI-reduced dosing or CNI- and/or steroid-sparing maintenance IS therapy [7–14]. Infection rates among SOT recipients who received limited alemtuzumab dosing are similar to those who received other lymphocyte depleting agents, such as antithymocyte globulin (ATG), for induction therapy [8, 15–18].

It is unknown whether SOT recipients receiving multiple doses of alemtuzumab, for both induction and maintenance therapy, would have increased infection rates. Between 2003 and 2005, the University of Minnesota pancreas and pancreas/kidney transplant program implemented a CNI- and steroid-free IS regimen, in which alemtuzumab was given as part of induction and maintenance therapy. The initial report evaluated the six-month noninfectious outcomes of this approach [19]. We studied the long-term infectious and noninfectious outcomes for recipients treated with this extended dosing of alemtuzumab compared to those in recipients treated without extended use of lymphocyte-depleting agents in the year before and after the extended alemtuzumab dosing period, with a more detailed description of the infectious complications.

## 2. Materials and Methods

### 2.1. Patients, Induction, and Maintenance of Immunosuppressive Therapies

Recipients of the simultaneous pancreas and kidney transplant (SPK), pancreas transplant alone (PTA), and pancreas after kidney transplant (PAK) from 2/28/2003 through 8/31/2005 received induction and maintenance IS therapy (CNI- and steroid-free) with alemtuzumab 30 mg intravenously (IV), referred to as extended alemtuzumab exposure (EAE). SPK received two doses of alemtuzumab, the first dose intraoperatively and the second on a postoperative day 2. PTA and PAK received alemtuzumab, intraoperatively and on postoperative days 2 and 4; in addition, ATG 1.25 mg/kg IV was given on a postoperative day 3. For all pancreas transplant sequence groups, the maintenance therapy for the first posttransplant year consisted of additional monthly doses of alemtuzumab (as long as the absolute lymphocyte count remains above 200/mm^3^) for total of 10 doses, and twice daily oral mycophenolate mofetil (MMF); see Table S1 in the Supplementary Material for comprehensive information. Recipients remained on CNI- and steroid-free after the first year of transplantation. However, a CNI was added if the recipient experienced an acute rejection. Recipients who did not receive induction therapy with alemtuzumab were excluded.

We compared outcomes of transplant recipients receiving EAE with those receiving ATG for induction IS plus the standard maintenance therapy before (1/1/2002 until 12/31/2002) and after (1/1/2006 until 12/31/2006) the implementation of EAE. Maintenance therapy was tacrolimus-based with a second agent—MMF or sirolimus, either with or without prednisone. Target tacrolimus levels were 10–15 ng/mL for the first 3 months following transplantation, then 8–10 ng/mL thereafter. Sirolimus target levels were 5–8 ng/mL. This group was named the standard induction and maintenance (SIM) IS therapy group. Recipients in the SIM group who received adjunctive doses of alemtuzumab for induction, rejection, or conversion (substituting alemtuzumab for CNI when patients developed CNI-associated nephropathy) therapy during the first year of transplant were excluded.

We studied outcomes of first pancreas transplant recipients (SPK, PTA, or PAK) receiving EAE vs. SIM therapy. If the kidney transplantation portion of a PAK occurred outside of the three above-mentioned chronological periods, they were included on the basis of the pancreas transplant date. The shortest follow-up for any transplanted patient was 138 months; hence, we truncated follow-up for all patients at 138 months.

### 2.2. Infection Prevention Standard Clinical Practice

Prevention of infection followed standard clinical practice for the time. Perioperative antibacterial prophylaxis was given with piperacillin-tazobactam for 3 days. In cases of penicillin allergy, imipenem-cilastatin was the alternate drug. Antifungal prophylaxis was with fluconazole for 7 days for SIM, and for 12 months for the EAE group. Tacrolimus dosages were adjusted when fluconazole was discontinued. For cytomegalovirus (CMV) prophylaxis, the SIM group received valganciclovir for 6 months if the donor/recipient (D/R) CMV serostatus was D+/R−, and for 3 months for R+. Valganciclovir dose was adjusted for the patient's creatinine clearance. The EAE group received valganciclovir for 12 months following transplantation regardless of the D/R CMV serostatus. Because we did not use CMV-reduced blood products during the transplant procedure, D-/R- recipients received valganciclovir for 3 months in the SIM group and 12 months in the EAE group. SIM and EAE groups received sulfamethoxazole/trimethoprim, almost indefinitely, for *Pneumocystis jirovecii* prophylaxis unless contraindicated.

### 2.3. Database

Demographic and transplant-related data was maintained on an Institutional Review Board (IRB) approved database. The Transplant Information Services (TIS) at the University of Minnesota prospectively collect demographic and transplant-related; professional data extractors perform this. We analyzed demographics, transplant-related data points, infection episodes, rejection events, and mortality up to 138 months after transplantation. Total (cold and warm) ischemic time was reported in minutes.

Infection data were collected cumulatively except for CMV infection, where only the first event was documented. The TIS did not collect data regarding signs or symptoms of CMV infection. Any CMV viremia that required therapy was defined as CMV infection. Bacterial infections caused by the same pathogen were considered unique events if they occurred ≥14 days apart. Similarly, fungal and viral infections were considered unique events if they occurred ≥84 days apart. When the pancreas antibody-mediated rejection (AMR) or acute cellular rejection (ACR) was diagnosed, it was assumed that this was associated with kidney rejection in SPK and PAK recipients. Except for a few patients with ACR that was diagnosed clinically, all AMR and most ACR cases were diagnosed by a pancreas biopsy. Pancreas graft failure was assessed for SPK, PTA, and PAK groups. Kidney graft failure was only assessed for the SPK group since for PAK recipients, the kidney graft may have been transplanted outside of the predetermined three chronological periods. If the death occurred with a functioning graft, this was not considered a graft loss. To assess the kidney function of all recipients (SPK, PTA, and PAK) in both groups, we calculated the mean creatinine at one year after transplant when this value was available. Recipients who ended up on dialysis within one year of transplant were analyzed separately. All cancer types were collected; however, only the first cancer event was counted for each patient when calculating the cancer rate. PTLD was reported as an independent outcome. The local IRB approved this retrospective study.

### 2.4. Statistical Analysis

All analyses considering contingency tables testing for association used the asymptotic chi-square test. To test a difference between the two protocol means on continuous variables, we used a *T*-test with an unequal variance assumption. For binary variables, such as counts of infections, we used a two-sample proportion test. For a majority of the aforementioned analyses, we used the package table one in R version 3.4.1. R packages survival and survminer were used to conduct the time to first event survival analyses and to yield related plots, respectively. For all of the survival models, we used the Cox proportional hazards model to estimate hazard ratios between the SIM and EAE groups. Survival models that only considered the protocol group as the predictor were labeled as “unadjusted,” while for models adjusting for sex, age, pancreatic duct drainage, and CMV serostatus were called “adjusted” models.

## 3. Results

Three hundred fifty-six patients underwent their first SPK, PTA, or PAK during the three distinct study periods. Eighteen patients were excluded for deviating from the induction and maintenance therapy protocol during a specific period (8 in SIM received alemtuzumab in addition to ATG, and 10 in EAE did not receive alemtuzumab for induction therapy) (see Table 1). Three hundred thirty-eight unique patients remained in the study. One hundred fifty-nine patients were in the SIM group (99/159 in 2002 and 60/159 in 2006) and 179 patients in the EAE group.

The 2 groups had similar demographics, characteristics, and transplant-related variables (Table 2). More recipients had pancreatic duct draining into the bladder (bladder drainage) in the SIM group (67.3%), while enteric drainage was more common in the EAE group (54%), *P* < 0.0001. CMV serostatus of the recipients in both groups was similar; however, the SIM group had less high risk recipients (D+/R−) compared to the EAE group, 20.6% vs. 32.0%, *P*=0.03.

Recipients in the EAE group received a mean ± SD of 5.2 ± 2.9 doses of alemtuzumab during the first year following transplantation.

### 3.1. Infectious Complications

#### 3.1.1. Overall Infections

**Figure 1:**
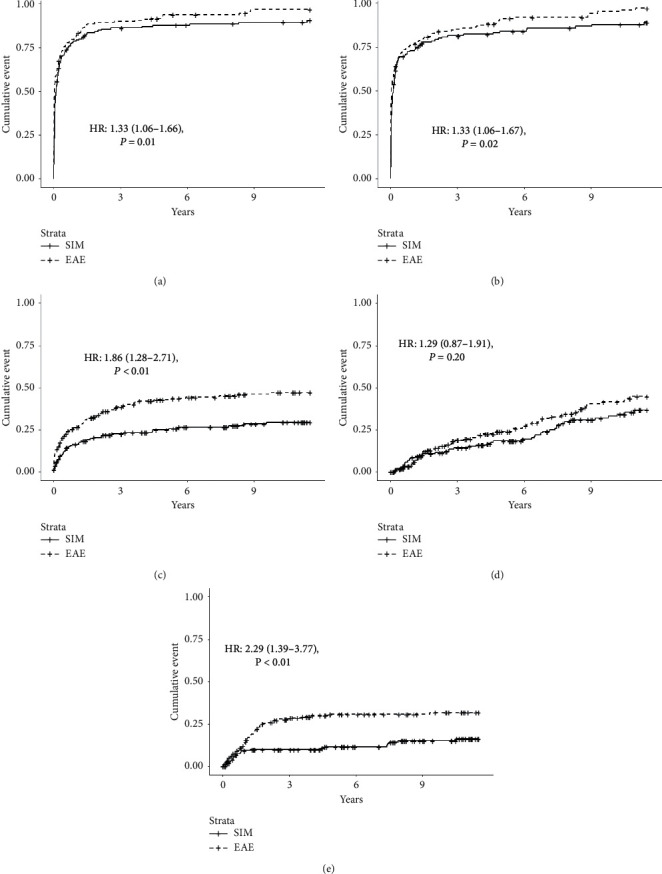
Cumulative incidence and unadjusted HR of infections among both groups. (a) All infections. (b) Bacterial infections. (c) Fungal infections. (d) Viral infections other than CMV. (e) CMV infections. EAE: extended alemtuzumab exposure; SIM: standard induction and maintenance therapy.

At the end of follow-up, the rate of overall infections was similar between patients who received SIM and those with EAE, 88.1% vs. 94.4%, respectively, *P*=0.06 (Table 3). However, the unadjusted hazard ratio (HR) for patients with EAE was 1.3 (95% CI 1.06–1.66; *P*=0.01) compared to those who received SIM therapy (Table 3 and Figure 1(a)). This higher risk of infection remained significant when HR was adjusted for recipient age, gender, pancreatic duct drainage, and CMV D/R serostatus (Table 3).

#### 3.1.2. Bacterial Infections

Over the 138-month follow-up, there were a total of 571 bacterial infection events in the SIM group affecting 135/159 (84.9%) and 964 in the EAE group affecting 166/179 (92.7%) patients (*P*=0.03). EAE recipients were at higher risk for bacterial infections (unadjusted HR 1.33 [95% CI, 1.06–1.67, *P*=0.02]; adjusted HR 1.37 [95% CI, 1.07–1.76, *P*=0.01]) (Table 3 and Figure 1(b))). Gram positive bacterial infection events developed at least once in 73% of SIM and 79% of EAE recipients, occurring more often than Gram negative infection events (42% of SIM and 49% of EAE recipients). *Clostridioides difficile* infection (CDI) occurred in 21 (13%) of SIM and 33 (18%) of EAE transplant recipients, *P*=0.25.

Since multiple doses of alemtuzumab would provide many months of lymphocyte depletion, mycobacterial endpoints were of special consideration. There was one (0.6%) mycobacterial infection in the SIM group (*Mycobacterium avium* complex (MAC) bacteremia occurring 6 years after transplantation). There were six (3.4%) infections in the EAE group (*P*=0.17 compared to SIM). Of these six cases, 2 recipients had bacteremia (one with *M. marinum* and one with MAC), 2 had lymph node involvement (one with a lymph node biopsy showing granuloma and another with a positive acid-fast bacilli staining), and 2 had pulmonary infections (one with *M. kansasii* and one with presumptive tuberculosis infection). Only two of these six infections occurred within one year of transplantation; the other four infections occurred between 19–82 months following transplantation (Table 3).

#### 3.1.3. Fungal Infections

There were 61 fungal infections affecting 42/159 (26.4%) recipients in the SIM group, and 131 infections affecting 77/179 (43.0%) recipients in the EAE group (*P*=0.002). The unadjusted HR (1.86 (95% CI, 1.28–2.71), *P* < 0.01) and adjusted HR (1.84 (95% CI, 1.24–2.74), *P* < 0.01) showed that patients with EAE were more likely to experience fungal infections than SIM patients (Table 3 and Figure 1(c)).

Fungal infections included mainly infections with *Candida* spp. (17% in SIM and 31% in EAE groups, *P*=0.005). Infections caused by *Aspergillus* spp. did not reach statistical significance (1.3% in SIM and 3.4% in EAE, *P*=0.37) (Table 3). In addition, one case of pulmonary histoplasmosis, two cases of *Pneumocystis jirovecii* pneumonia (PJP), and two patients with cryptococcal infection were observed, all in the EAE group.

#### 3.1.4. Viral Infections

There were 67 non-CMV viral infection events in 44/159 (27.7%) in the SIM group and 77 infection events in 59/179 (33.1%) patients in the EAE group, (*P*=0.35). Both groups had a similar risk of experiencing non-CMV viral infection with similar unadjusted and adjusted HR for both groups (Table 3 and Figure 1(d).

Since the information about CMV infections accounted only for the first CMV infection event, we do not have a total of CMV events in either group. CMV infections occurred in 22 (13.8%) patients in the SIM group and 51 (28.5%) in the EAE group, *P*=0.002. The unadjusted HR (2.29 (95% CI, 1.39–3.77, *P* < 0.01) and adjusted HR (1.95 (95% CI, 1.16–3.26), *P*=0.01) were significantly higher for those who had EAE, compared to SIM therapy, as shown in Table 3 and Figure 1(e). The mean time ± SD from the date of transplantation to the first CMV infection event was 958.1 ± 1215.9 days for the SIM group and 505.2 ± 548.9 days for the EAE, *P*=0.11. According to the CMV universal prophylaxis protocol applied for each group, the mean time ± SD from the last dose of valganciclovir to the first CMV event was 817.1 ± 1223.8 days for the SIM group and 140 ± 548.9 days for the EAE group, *P*=0.02 (Table 4).

### 3.2. Rejection Complications and Allograft Failure

**Figure 2:**
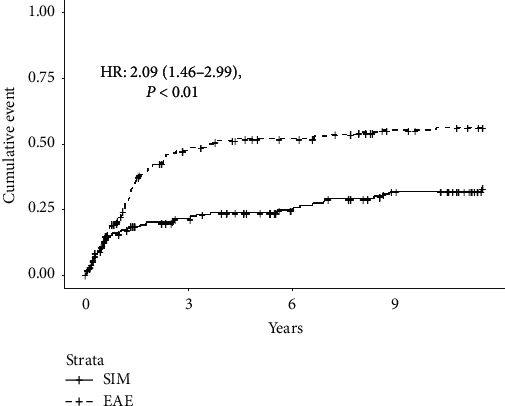
Cumulative incidence of cellular mediated rejection. EAE: extended alemtuzumab exposure; SIM: standard induction and maintenance therapy.

During the 138 months following each unique transplantation, there were only two pancreas AMR events occurring in two patients, one in each group. On the other hand, there were a total of 213 events of pancreas ACR. Sixty-nine events of ACR occurred in 45 (28.3%) unique patients in the SIM group, and 144 events in 89 (49.7%) patients in the EAE, *P* ≤ 0.001 (Table 3). The unadjusted HR (2.09, 95% CI 1.46–2.99 (*P* < 0.01)) and adjusted HR (2.29, 95% CI 1.57–3.33 (*P* < 0.01)) showed a higher risk for ACR in those with EAE (Table 3 and Figure 2).

**Figure 3:**
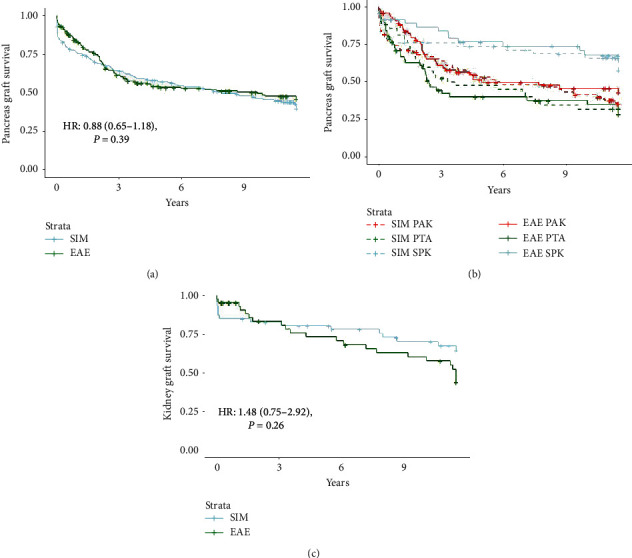
Kaplan–Meier plot of pancreas and kidney allograft survival. (a) Pancreas allograft failure. (b) Pancreas allograft failure according to transplant order. (c) Kidney allograft failure among those who received SPK only. EAE: extended alemtuzumab exposure; PAK: pancreas after kidney; PTA: pancreas transplant alone; SIM: standard induction and maintenance therapy; SPK: simultaneous pancreas and kidney.

At the end of follow-up, the pancreas allograft failed in 88 (55.3%) in the SIM group and 85 (47.5%) of patients the EAE group (*P*=0.182) with HR 0.88 (0.65–1.18), *P*=0.39. The kidney allograft in SPK recipients failed in 15/47 (31.9%) in the SIM group and 22/47 (46.8%) in the EAE (*P*=0.20), HR: 1.48 (0.75–2.92), *P*=0.26 (see Table 3 and Figure 3).

### 3.3. Other Outcomes

#### 3.3.1. Cancer excluding PTLD

During the 138-month follow-up period, 46 (28.9%) in the SIM group developed cancer and 54 (30.2%) in the EAE group (*P*=0.89). Skin cancer was the most common form affecting 32 (20.1%) in the SIM group and 42 (23.5%) in the EAE group (*P*=0.54). The most common form of skin cancer was squamous cell carcinoma (SCC), affecting total 46 patients (17 in the SIM Group and 29 in the EAE group), followed by basal cell carcinoma (BCC) in a total of 31 patients (13 and 18, respectively), and five melanoma (2 and 3, respectively). There were many other types of skin cancers including carcinoma in situ, dysplasia, and sarcoma.

Cancer types other than skin cancer and PTLD included 20 patients with gynecological (8 in the SIM group and 12 in the EAE group), six patients with gastrointestinal (2 and 4, respectively), 6 urogenital (3 in each group), and two endocrine cancers (both in the SIM group).

#### 3.3.2. PTLD

There were 10 transplant recipients who received the diagnosis of PTLD during the 138-month follow-up period. Three recipients in the SIM group (1.9%) and seven in the EAE group (3.9%) (*P*=0.44) developed PTLD at a mean ± SD of 919 ± 1357.3 days and 1818.6 ± 1881.3 days from the day of transplantation, respectively (*P*=0.34 95% CI−3556.6–1758.1).

#### 3.3.3. Kidney Function

The kidney function of all recipients was evaluated by assessing the creatinine value at one year after transplant and whether the patient ended up on dialysis within one year of transplant. There was no difference in the kidney function between SIM and EAE groups when it comes to mean creatinine value at one year after transplant (1.5 for both groups, *P*=0.89) and those who ended up on dialysis within one year of transplant (4.5% in SIM vs. 2.3% in EAE, *P*=0.43).

#### 3.3.4. Survival

**Figure 4:**
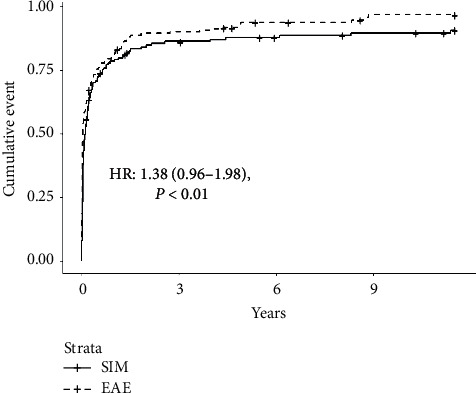
Cumulative incidence of survival. EAE: extended alemtuzumab exposure; SIM: standard induction and maintenance therapy.

There were 121 transplant recipients who died during the follow-up period. The death rate in the SIM group (31%) and EAE group (40%) were similar, *P*=0.09. The risk of death was similar as well between both groups (adjusted HR 1.38 (0.96–1.98), *P*=0.08 and adjusted HR 1.31 (0.89–1.92), *P*=0.17) (see Table 3 and Figure 4).

## 4. Discussion

Following SOT procedures, maintenance IS therapy is essential for graft survival and typically includes a CNI (usually tacrolimus), MMF, and prednisone. This, however, is associated with multiple adverse events. So many studies have looked to determine an induction and maintenance IS that would avoid such events without jeopardizing the allograft. In kidney transplant alone (KTA), the induction with ATG followed by steroid-free maintenance IS, achieved a graft survival rate similar to that of historical KTA recipients who received the triple maintenance IS with fewer CMV infections, posttransplant diabetes cataract, and avascular necrosis when compared to historical controls with the same induction IS but receiving the triple maintenance IS [20, 21]. Similarly, steroid-free maintenance IS, following induction with alemtuzumab, was associated with favorable graft and adverse events outcomes in KTA [8, 9, 11, 12, 14] and SPK recipients [12, 15, 22–26] compared to induction with ATG or nonlymphocytes depleting agents, with either triple or prednisone-free maintenance IS. In these studies, the total alemtuzumab doses were 40–60 mg given in 20–30 mg/day. The infectious and noninfectious outcomes after EAE (higher than a total of 40–60 mg) with CNI- and prednisone-free maintenance IS have never been described before.

When compared to the SIM, we documented higher risks of overall, bacterial, fungal, and CMV infections after PTA, SPK, and PAK with CNI- and steroid-free and EAE therapy over 12 months after transplantation; in contrast to prior studies in which extended alemtuzumab was not used. In SPK recipients who received the triple maintenance IS following induction with alemtuzumab, Pascual et al. reported rates of opportunistic infections similar to those who received basiliximab up to one year of follow-up (65% vs. 58%, respectively, *P*=0.61) [27]. Bank et al. compared SPK recipients with alemtuzumab induction and steroid-free maintenance IS to those with ATG induction and triple maintenance IS with a 3-year follow-up [22]. There was no difference in the rates of EBV (4% each, respectively *P*=0.85), wound (7% vs. 8%, respectively *P*=0.74), abdominal (16% vs. 24%, respectively *P*=0.27), and pulmonary (6% vs. 9%, respectively *P*=0.25) infections. Donor derived (15% in alemtuzumab vs. 16% in ATG, *P*=0.81) and reactivation (3% in alemtuzumab vs. 5% in ATG, *P*=0.52) of CMV infection rates were also similar between both groups. Only urinary tract infections were more common in the ATG group (49% vs. 80%, respectively *P*=0.00.1) [22].

Among SPK receiving steroid-free maintenance IS, regardless of the induction IS, studies have shown similar findings. Reddy et al. compared the outcomes of a rapid withdrawal of steroids in SPK recipients who received alemtuzumab to those who received ATG for induction; the CMV infection rate was similar in both groups (10% vs. 18%, respectively *P*=NS), slightly favoring alemtuzumab [25]. Stratta et al. also compared steroid-free maintenance IS in SPK receiving alemtuzumab to those receiving ATG for induction at 5 years after transplantation and found that the rate of major infections was similar in both groups (39% vs. 67%, respectively *P*=0.13), also favoring alemtuzumab [15]. In addition, no CMV infections were documented in the alemtuzumab group compared to 17% in the ATG group, *P*=0.05 [15]. A study by Kaufman et al. reported over a 3-yearly follow-up a higher cumulative incidence of viral infections in SPK recipients with steroid-free maintenance IS with ATG for induction (∼40%) compared to those who received alemtuzumab (∼10%), *P*=0.007 [24]. The CMV infection rate was higher in the ATG group (19%) vs. alemtuzumab group (6%), but it did not reach a statistical significance, *P*=0.11 [24].

Our PTA, SPK, and PAK recipients with CNI- and steroid-free maintenance IS received an EAE with up to 10 doses of alemtuzumab with a mean (±SD) of 5 (± 3) doses. This is substantially higher than the maximum total of 60 mg of alemtuzumab given in these previously described studies. We believe that the high dose of alemtuzumab explains the high risk of overall (HR 1.3 (1.1–1.7), *P*=0.01) and bacterial infections (HR 1.3 (1.1–1.7), *P*=0.02). It also explains why these patients were almost twice as likely to develop fungal (HR=1.9 (1.3–2.7), *P* < 0.01) and CMV infections (HR=2.3 (1.4–3.8), *P* < 0.01) compared to patients receiving the SIM therapy. These higher risks of the overall, bacterial, and specifically fungal and CMV infections remained significant when adjusted for age, gender, pancreatic duct drainage, and CMV serostatus as shown in Table 3. It is interesting that one study by Magliocca et al. described a significantly increased rate of CMV infection in SPK recipients receiving a total of 60 mg of alemtuzumab in two divided doses for induction with steroid-free maintenance (29%) compared to those who received ATG for induction with the triple maintenance IS (16%), *P*=0.002 [28]. This has led the authors to change their protocol to consist of a single 30 mg dose of alemtuzumab. Also, though in a nonpancreas transplant population, Margreiter et al. showed that KTA with alemtuzumab induction and tacrolimus-only maintenance IS were twice as likely to experience CMV infection (relative risk 2.3 (1.1–4.9)) compared to those who received tacrolimus and steroid preoperatively followed by the triple maintenance IS [11]. These findings further corroborate that the risk of infections seems to proportionately correlate with the dose of alemtuzumab.

Though the long-term follow-up in our study may have resulted in the detection of more infectious events, we believe this effect was negligible, as both study groups truncated follow-up at the shortest individual patient noncensured follow-up duration. In addition, Bosmuller et al., who reported the outcomes of SPK recipients after a follow-up of longer than 9 years after transplantation, have not detected more infectious events in those who received alemtuzumab for induction followed by tacrolimus-only maintenance IS, compared to those with ATG for induction and the triple maintenance IS [23]. The number of patients was very small, however, compared to our study (14 in the alemtuzumab group and 16 in ATG) [23].

CMV infection is the most common opportunistic infection in SOT recipients with a great impact on morbidity and mortality [29]. In our study, the EAE was not only associated with a high risk for CMV infection but also with an earlier occurrence after the discontinuation of the CMV universal prophylaxis (mean 140 ± 549 days) compared to SIM therapy (mean 817 ± 1224 days). This early occurrence of the CMV infection was consistent in all CMV D/R serostatus subgroups. It is plausible that our patients with EAE need longer than one year of CMV prophylaxis, given the multiple dosing regimen of alemtuzumab. However, this may not be feasible due to adverse events and financial expenses incurred by the patient and the health system.

Among the bacterial infections, we documented seven mycobacterial infections with no statistically significant difference in their distribution between both groups. It was interesting, however, that mycobacterial infections occurred only in one patient (0.6%) in the SIM group, similar to rates reported in the literature of 0.5–1% [30, 31]. In the EAE group, however, a higher rate of mycobacterial infection (3%) likely due to the extended lymphopenia associated with the EAE. Four of these six infections occurred 19–82 months after transplantation. The other two occurred within one year of transplantation. Hence, it is reasonable to have a heightened level of suspicion for opportunistic mycobacterial infections years after receiving high cumulative doses of alemtuzumab.

To our knowledge, there is no available literature to suggest that the use of alemtuzumab is associated with CDI. However, the use of ATG is considered a risk factor for CDI [32]. Historically, the rate of CDI among pancreas and/or kidney transplant recipients is up to 5%, similar to the rate in the general population [33–35]. The rate of CDI in our study was similarly high in SIM (13%) and EAE (18%), *P*=0.25. The reason behind this high rate of CDI is beyond the scope of this study.

Among fungal infections, candidal infections were the most common (Table 3); this is consistent with findings of prior studies [30, 31, 36]. For example, Descourouez et al. found that KTA recipients who received alemtuzumab for more than one KTA procedure had higher rates of fungal infections (47%) when compared to those who received ATG for the second KTA after receiving alemtuzumab for the original KTA (11%), *P*=0.02 [37]. In addition, we found that all other major opportunistic fungal infections of pulmonary histoplasmosis (*n* = 1), PJP (*n* = 2), and cryptococcosis (*n* = 2) occurred in the EAE group. PJP has been reported with alemtuzumab used in the treatment of patients with HM [2]. No prior studies reported PJP infections in SOT recipients receiving single dose alemtuzumab [8, 10, 13, 23, 30, 31].

ACR was twice as likely to occur in the EAE group. These findings contradict others by Pascual et al. who found that among SPK recipients, an induction with two doses of alemtuzumab was associated with a lower rate of ACR (3%) when compared to basiliximab (15%), *P*=0.02 but was not protective against AMR with a rate of 18% vs. 14%, *P*=0.6 [27]. Most of the other studies evaluating the effectiveness of steroid-free maintenance IS following induction with alemtuzumab did not differentiate AMR and ACR, and found similar [15, 23–26, 28] or better [22] rejection and graft survival rates when compared to the conventional IS. When our group reported their 6-month experience with the EAE and CNI- and steroid-free maintenance IS, we noted higher rates of graft loss due to rejection in PTA (15% vs. 3%, *P*=0.06) and a higher rate of first reversible rejection in SPK (41% vs. 9%, *P*=0.0003), compared to PTA and SPK in a historical SIM group, respectively [19]. The overall rejection and graft survival rates were similar between both groups at six months, however [19]. Although, in this long-term follow-up study, we did not look at the rejection or graft survival rates in relation to the transplant order (PTA, PAK, SPK), it is interesting that we showed a higher risk of ACR in the EAE group. This is likely due to the long-term follow-up during a period where the alemtuzumab protective effect is waning combined with the lack of CNI and steroid use, at least within the first year. Indeed Banks et al. found that the majority of rejection in SPK recipients in the alemtuzumab induction group had late rejections compared to those in the ATG induction group [22]. In our study, both the kidney and pancreas graft survival were similar among groups, as was the kidney function. However, evaluating whether CNI- and steroid-free maintenance offered additional protection to the kidney function was beyond the scope of this study.

There were only 10 cases of PTLD, three in the SIM group (1.9%) and seven (3.9%) in the EAE group, *P*=0.44. The lack of statistical significance is likely due to the small number of patients with PTLD. It is interesting however that the PTLD incidence was double in the EAE and occurred sooner and it is worth noting.

Our study has the unique opportunity to evaluate different outcomes of EAE, with up to 10 doses of alemtuzumab given within the first year following pancreas transplantation, with long-term follow-up. Our study has limitations including the inherent shortcomings of a single-center, retrospective study. We depended on the database of the TIS in the University of Minnesota, so we were not able to confirm the accuracy of these data. In addition, our patients may have received additional doses of alemtuzumab or ATG later during the long-term follow-up period that we did not account for during our evaluation. We expect that during the 138-month follow-up, CNI was added to the maintenance IS therapy of most patients which we did not account for. However, our analysis is similar to the intention to treat analysis in prospective studies. For ease of computation, we selected a time-till-first-event analysis for infections, cancers, and rejections, which omitted information potentially gained by incorporating repeated outcomes. Additionally, a dose-outcome relationship could be evaluated if we allowed the number of alemtuzumab doses to be a time-varying covariate in the survival model.

## 5. Conclusion

The cumulative alemtuzumab dosing is associated with higher risks of overall, bacterial, fungal (mainly candidal), and CMV infections. CMV infections occurred significantly earlier after the discontinuation of the universal CMV prophylaxis in the EAE group than the SIM group. There were more opportunistic infections in the EAE group including PJP, histoplasmosis, cryptococcosis, and mycobacterial infections. Our study adds to the body of literature that demonstrates that the rate of infections increases when a greater number of doses of alemtuzumab are used [3–6, 11, 28, 37, 38]. Although CNI and steroids are associated with adverse events, it seems that an EAE and CNI- and steroid-free maintenance IS would not be a safe alternative to avoid these adverse events.

## Figures and Tables

**Table 1:** Selection criteria and patients inclusion.

Selection criteria	Number of patients excluded	Number of patients remained			Total
		SIM 2002	EAE 2003–2005	SIM 2006	
First pancreas transplant		106	189	61	356
Alemtuzumab for conversion^*∗*^	4	102	189	61	352
Alemtuzumab for rejection treatment in the first year after transplant	3	99	189	61	349
Alemtuzumab for induction	1	99	189	60	348
ATG for induction	8	99	181	60	340
Methylprednisone for induction	1	99	180	60	339
Daclizumab for induction	1	99	179	60	338

Study group		SIM	EAE		Total
		159	179		338

^*∗*^Conversion therapy was defined as the need to substitute alemtuzumab for calcineurin inhibitor(s) due to calcineurin inhibitor-associated nephropathy. ATG, antithymocyte globulin; EAE: extended alemtuzumab exposure; SIM: standard induction and maintenance.

**Table 2:** Characteristics, demographics, and distribution of patients according to the induction and maintenance therapy received after transplantation.

Variables	Standard induction and maintenance therapy *n* = 159	Extended alemtuzumab exposure *n* = 179	*P* value
Race (%)			0.42
Caucasians	157 (98.7)	173 (96.6)	
Native Americans	1 (0.6)	4 (2.2)	
African Americans	1 (0.6)	2 (1.1)	
Age at transplant, years (mean ± SD)	43.03 ± 8.68	43.60 ± 8.76	0.54
Recipient gender, male (%)	86 (54.1)	87 (48.6)	0.37
Primary disease (%)			0.42
Diabetes type 1	151 (95.0)	164 (91.6)	
Diabetes type 2	5 (3.1)	11 (6.1)	
Native pancreatectomy	3 (1.9)	4 (2.2)	
Transplant order (%)			0.59
PAK	70 (44.0)	76 (42.5)	
PTA	42 (26.4)	56 (31.3)	
SPK	47 (29.6)	47 (26.3)	
Pancreatic duct drainage^a^			<0.0001
Bladder drainage	105 (67.3)	78 (44.8)	
Enteric drainage	50 (32.1)	94 (54)	
Other	1 (0.6)	2 (1.15)	
Donors' cause of death (%)			^b^0.40
Cardiovascular	1 (0.6)	2 (1.1)	
Cerebrovascular	36 (22.6)	45 (25.1)	
Trauma	67 (42.1)	120 (67.0)	
Unknown	55 (34.6)	12 (6.7)	
ECD (%)	1 (1.0)	3 (1.7)	1.00
DCD (%)	5 (3.2)	8 (4.5)	0.72
Total ischemic time^c^, minutes (mean ± SD)	1082.78 ± 306.36	1050.01 ± 281.92	0.33
History of transplant prior to 2002 (%)	34 (21.4)	34 (19.0)	0.68
Time to retransplant, days (mean ± SD)	1935.26 ± 1760.39	1944.76 ± 1310.70	0.98
Length of stay^d^, days (mean ± SD)	13.14 ± 14.83	14.41 ± 12.29	0.39
CMV serostatus^e^ (%)			0.07
D+/R−^f^	32 (20.6)	57 (32.0)	
R+	94 (60.6)	94 (52.8)	
D−/R−	29 (18.7)	27 (15.2)	
Gender D/R match (%)	91 (57.2)	90 (50.3)	0.24

^a^Data available for 156/159 in the standard induction and maintenance therapy group, and 174/179 in the extended alemtuzumab exposure group. ^b^Chi-Square Test computed by omitting the unknowns. ^c^Total ischemic time is the summation of both cold and warm ischemic time. ^d^Length of stay at the time of transplantation. ^e^Data are available for 155/159 for the standard induction and maintenance therapy group and 178/179 for the extended alemtuzumab exposure group. ^f^*P*=0.03. DCD: donation after cardiovascular death; D/R: donor/recipient; ECD: expanded criteria donors; PAK: pancreas after kidney transplant; PTA: pancreas transplant alone; SD: standard deviation; SPK: simultaneous pancreas and kidney transplant.

**Table 3:** Infection, rejection, graft failure, cancer, and mortality outcomes among pancreas transplant recipients receiving the standard induction and maintenance therapies vs. those with extended alemtuzumab exposure.

Outcomes^a^	Standard induction and maintenance therapy *n* = 159	Extended alemtuzumab exposure *n* = 179 (%)	*P* value	Unadjusted HR (95% CI)	*P* value	Adjusted HR^b^ (95% CI)	*P* value
All infections^c^	140 (88.1)	169 (94.4)	0.06	1.33 (1.06–1.66)	0.01	1.41 (1.11–1.79)	0.01
Bacteria^c^	135 (84.9)	166 (92.7)	0.03	1.33 (1.05–1.67)	0.02	1.37 (1.07–1.76)	0.01
Gram positive	116 (73.0)	143 (79.9)	0.17				
*C difficile*	21 (13.2)	33 (18.4)	0.25				
Gram negative	67 (42.1)	88 (49.2)	0.24				
Mycobacteria	1 (0.6)	6 (3.4)	0.17				
Fungi^c^	42 (26.4)	77 (43.0)	0.002	1.86 (1.28–2.71)	<0.01	1.84 (1.24–2.74)	<0.01
*Aspergillus* spp.	2 (1.3)	6 (3.4)	0.37				
*Candida* spp.	27 (17.0)	55 (30.7)	0.005				
Other	21 (13.2)	32 (17.9)	0.30				
Non-CMV viruses^c^	44 (27.7)	59 (33.1)	0.35	1.29 (0.87–1.91)	0.20	1.24 (0.82–1.86)	0.31
HSV/VZV	24 (15.1)	40 (22.3)	0.12				
Respiratory viruses	10 (6.3)	5 (2.8)	0.19				
BKV	5 (3.1)	7 (3.9)	0.93				
Other non-CMV viruses^c^	18 (11.3)	16 (8.9)	0.59				
CMV	22 (13.8)	51 (28.5)	0.002	2.29 (1.39–3.77)	<0.01	1.95 (1.16–3.26)	0.01
Other infections^e^	5 (3.1)	12 (6.7)	0.21				
ACR	45 (28.3)	89 (49.7)	<0.001	2.09 (1.46–2.99)	<0.01	2.29 (1.57–3.33)	<0.01
Pancreas allograft failure	88 (55.3)	85 (47.5)	0.18	0.88 (0.65–1.18)	0.39	0.84 (0.61–1.15)	0.28
PAK	42 (26.4)	38 (21.2)	0.32				
PTA	29 (16.2)	34 (21.4)	0.97				
SPK	17 (10.7)	13 (7.3)	0.36				
Kidney allograft failure^f^	15 (31.9)	22 (46.8)	0.14				
Kidney function of all recipients^g^							
Creatinine (mean ± SD)^h^	1.52 ± 1.33	1.49 ± 0.89	0.89				
Dialysis within one year of transplant	7 (4.5)	4 (2.3)	0.43				
Cancer	46 (28.9)	54 (30.2)	0.89	1.40 (0.72–2.72)	0.32	1.34 (0.67–2.66)	0.41
Skin cancer	32 (20.1)	42 (23.5)	0.54	1.33 (0.84–2.10)	0.23	1.24 (0.76–2)	0.39
Death	49 (30.8)	72 (40.2)	0.09	1.38 (0.96–1.98)	0.08	1.31 (0.89–1.92)	0.17

^a^First event. ^b^Adjusted for patients' age, gender, pancreatic duct drainage, and CMV serostatus. ^c^Each unique patient may have more than one infection. ^d^Human papillomavirus, viral meningitis or encephalitis, hepatitis C virus, gastrointestinal viruses, and EBV. ^e^Not specified. ^f^Of 47 patients in each group who received SPK. ^g^Data available for 157/159 in the standard induction and maintenance therapy group, 174/179 in the extended alemtuzumab group. ^h^Excluding those who had to go on dialysis within one year of transplant. ACR: acute cellular rejection; BKV: BK virus; CI: confidence interval; HR: hazard ratio; HSV/VZV: herpes simplex virus/varicella zoster virus; PAK: pancreas after kidney transplant; PTA: pancreas transplant alone; SD, standard deviation; SPK: simultaneous pancreas and kidney transplant.

**Table 4:** Characteristics of patients with CMV infections and timing of CMV infections in relation to transplantation and the universal prophylaxis protocol.

Group CMV D/R	Patients with CMV infection	Days from transplant till first event (mean ± SD)	Days from the end of the universal prophylaxis to first event, mean ± SD
Standard induction and maintenance therapy	22	958.1 ± 1215.9^a^	817.1 ± 1223.8^b^
D+/R−	7	508.4 ± 983.9	325.9 ± 983.9
R+	15	1167.9 ± 1286.3	1046.3 ± 1286.3

Extended alemtuzumab exposure	51^c^	505.2 ± 548.9^a^	140.2 ± 548.9^b^
D+/R−	25	552.0 ± 661.7	187.0 ± 661.7
R+	25	474.1 ± 424.3	109.1 ± 424.3

Patients in the standard induction and maintenance therapy group received valganciclovir for 6 months if the CMV serostatus was D+/R−, and for 3 months if R+ or D−/R−. All patients in the extended alemtuzumab exposure group received valganciclovir for 12 months following transplantation. D/R: donor/recipient CMV serostatus; SD: standard deviation. ^a^Comparing these two values, *P*=0.11. ^b^Comparing these two values, *P*=0.02. ^c^There was one D−/R− transplant recipient in the extended alemtuzumab exposure group.

## Data Availability

Data used to support this study are included within this manuscript. If additional data are needed, they would be available upon request from the corresponding author.

## Supplementary Materials

Supplementary Materials Table S1: standard immunosuppression during the calcineurin inhibitor- and steroid-free period.
