# Supplementary material for: Long-Term Infectious and Noninfectious Outcomes of Monthly Alemtuzumab as a Calcineurin Inhibitor- and Steroid-Free Regimen for Pancreas Transplant Recipients
Source: Can J Infect Dis Med Microbiol. 2020 Oct 9;2020:8883183. doi: 10.1155/2020/8883183 (PMC7569440; doi:10.1155/2020/8883183)
Supplement: Supplementary Materials — Table S1: standard immunosuppression during the calcineurin inhibitor- and steroid-free period. [file 8883183.f1.docx]

Table S1. Standard immunosuppression during the CNI- and steroid-free period.

| Simultaneous Pancreas Kidney | Pancreas After Kidney | Pancreas Transplant Alone |
| --- | --- | --- |
| **Alemtuzumab:**  30 mg IV intraoperatively  30 mg IV on POD #2  30 mg IV monthly to a  total of 8 doses in 1^st^ year  (only if ALC ≥200/cu mm)  Give Solu-Medrol 500 mg before 1^st^ dose and 100 mg before each subsequent dose  **Mycophenolate mofetil**:  Start posteratively 1 gram po twice daily    *Mycophenolate mofetil dosing guide*:  Absolute Neutrophil Count (ANC)  <2.5 2 gram/day  <2 1 gram/day  <1.5 0.5 gram/day  <1 hold  For ANC < 1.5 give GCSF 300mcg sq daily x 3  If Mycophenolate mofetil dose is <1 gram/day for gastrointestinal toxicity, add Rapamune 2 mg/day, target level up to 8-12 ng/ml depending on Mycophenolate mofetil dose | *If on Kidney Steroid Avoidance Study (simultaneous study during the same period):*  **Alemtuzumab**:  30 mg IV intraoperatively  30 mg IV on POD #2  Give Solu-Medrol 500 mg before 1^st^ dose and 100 mg before each subsequent dose  Continue that protocol’s maintenance immunosuppression regimen – Tacrolimus/Rapamune or Cyclosporine/Mycophenolate mofetil  *If not on above study:*  **Alemtuzumab:**  30 mg IV intraoperatively  30 mg IV on POD #2 and #4  30 mg IV monthly to a  total of 8 doses in 1^st^ year  (only if ALC ≥200/cu mm)  Give Solu-Medrol 500 mg before 1^st^ dose and 100 mg before each subsequent dose  **Thymoglobulin**:  1.25 mg/kg IV on POD # 3  Give Solu-Medrol 100 mg IV prior to dose  **Mycophenolate mofetil**: Start posteratively 1 gram po twice daily  Use dosing guide from column 1 | **Alemtuzumab:**  30 mg IV intraoperatively  30 mg IV on POD #2 and #4  30 mg IV monthly to a  total of 8 doses in 1^st^ year  (only if ALC ≥200/cu mm)  Give Solu-Medrol 500 mg before 1^st^ dose and 100 mg before each subsequent dose  **Thymoglobulin**:  1.25 mg/kg IV on POD # 3  Give Solu-Medrol 100 mg IV prior to dose  **Mycophenolate mofetil**: Start posteratively 1.5 gram po twice daily  Use dosing guide from column 1 |
|  |  |  |
|  |  |  |
|  |  |  |
|  |  |  |

CNI, calcineurin inhibitors; IV, intravenous; POD, postoperative day; ALC = absolute lymphocyte count
